# Tolvaptan activates the Nrf2/HO-1 antioxidant pathway through PERK phosphorylation

**DOI:** 10.1038/s41598-019-45539-8

**Published:** 2019-06-25

**Authors:** Tamami Fujiki, Fumiaki Ando, Kana Murakami, Kiyoshi Isobe, Takayasu Mori, Koichiro Susa, Naohiro Nomura, Eisei Sohara, Tatemitsu Rai, Shinichi Uchida

**Affiliations:** Department of Nephrology, Tokyo Medical and Dental University (TMDU), Tokyo, Japan

**Keywords:** Cell signalling, Chronic kidney disease

## Abstract

Tolvaptan, a vasopressin type 2 receptor antagonist initially developed to increase free-water diuresis, has been approved for the treatment of autosomal dominant polycystic kidney disease in multiple countries. Furthermore, tolvaptan has been shown to improve the renal functions in rodent models of chronic kidney disease (CKD); however, the underlying molecular mechanisms remain unknown. CKD is characterized by increased levels of oxidative stress, and an antioxidant transcription factor—nuclear factor erythroid 2-related factor 2 (Nrf2)—has been gaining attention as a therapeutic target. Therefore, we investigated the effects of tolvaptan and a well-known Nrf2 activator, bardoxolone methyl (BARD) on Nrf2. To determine the role of tolvaptan, we used a renal cortical collecting duct (mpkCCD) cell line and mouse kidneys. Tolvaptan activated Nrf2 and increased mRNA and protein expression of antioxidant enzyme heme oxygenase-1 (HO-1) in mpkCCD cells and the outer medulla of mouse kidneys. In contrast to BARD, tolvaptan regulated the antioxidant systems via a unique mechanism. Tolvaptan activated the Nrf2/HO-1 antioxidant pathway through phosphorylation of protein kinase RNA-like endoplasmic reticulum kinase (PERK). As a result, tolvaptan and BARD could successfully generate synergistic activating effects on Nrf2/HO-1 antioxidant pathway, suggesting that this combination therapy can contribute to the treatment of CKD.

## Introduction

Tolvaptan is a highly selective and orally effective vasopressin type 2 receptor (V2R) antagonist that inhibits vasopressin-mediated water reabsorption in the kidney and promotes free-water diuresis in patients with heart failure and with syndrome of inappropriate secretion of antidiuretic hormone (SIADH)^1,2^. In addition, tolvaptan is effective in suppressing renal cyst growth in patients with autosomal dominant polycystic kidney disease (ADPKD)^3^. These beneficial effects of tolvaptan are primarily caused by the inhibition of intracellular cyclic adenosine monophosphate (cAMP) productions^4,5^. Moreover, tolvaptan has been shown to reduce proteinuria and improve renal function in rodent models of CKD. Tolvaptan improves morphologic change of podocyte and reduces proteinuria and serum creatinine in a rodent model of puromycin aminonucleoside induced-nephrosis^6^. Tolvaptan also ameliorates interstitial fibrosis and creatinine clearance in a rodent model of heart failure^7^. However, the underlying molecular mechanisms of renal protection by tolvaptan remain unclear.

Oxidative stress is crucially involved in the development and progression of CKD^8^. Nuclear factor erythroid 2-related factor 2 (Nrf2) is the key transcription factor that regulates antioxidant defense systems in response to oxidative stress. Under basal conditions, Nrf2 is sequestered in the cytoplasm via binding to Kelch-like ECH-associated protein 1 (Keap1)^9^. During exposure to oxidants, the interaction between Keap1 and Nrf2 is disrupted, following which Nrf2 is translocated to the nucleus, where it increases the transcription of antioxidant enzymes, such as heme oxygenase-1 (HO-1)^10,11^. In rodent models of CKD, an overt increase of oxidants, including superoxide and hydrogen peroxide, is observed^12^. Nevertheless, Nrf2 nuclear translocation is impaired, resulting in unresponsiveness of antioxidant enzymes to oxidative stress^13,14^. Nrf2-dysregulation in CKD produces a vicious cycle of increasing oxidative stress and renal damage^15^. Conversely, the activation of Nrf2 has been gaining attention as a therapeutic target. An Nrf2 activator, bardoxolone methyl (BARD), has been demonstrated to ameliorate kidney functions in patients with CKD in several clinical trials (TSUBAKI; NCT02316821)^16,17^.

Therefore, in the present study, we focused on the effect of tolvaptan on Nrf2. We hypothesized that tolvaptan leads to Nrf2 nuclear translocation and induces HO-1 expression as with BARD. Moreover, we assumed that tolvaptan and BARD additionally or synergistically activate the Nrf2/HO-1 antioxidant pathway.

## Results

### Tolvaptan induces Nrf2 nuclear translocation and HO-1 expression in mpkCCD cells

**Figure 1:**
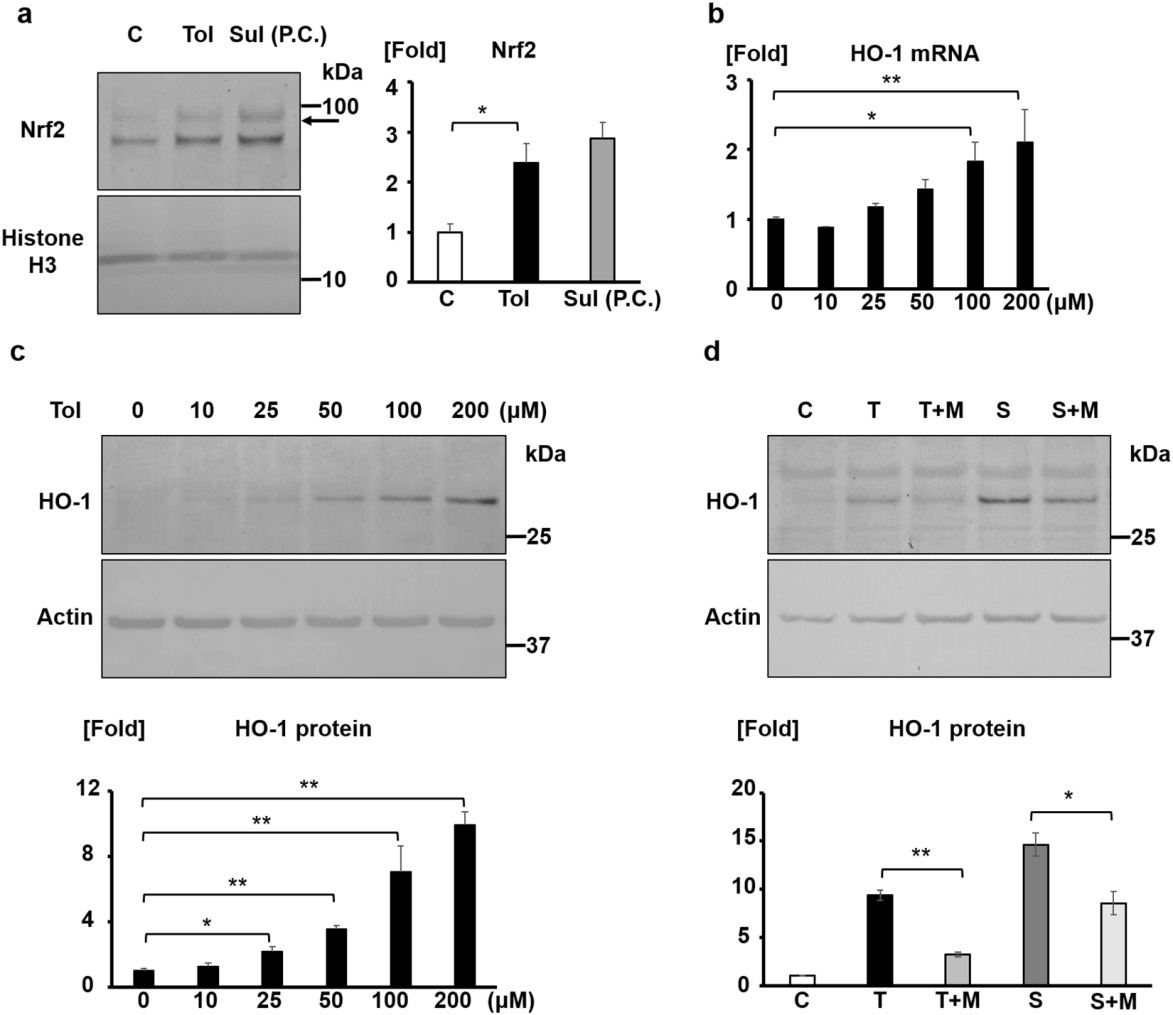
Tolvaptan induces Nrf2 nuclear translocation and HO-1 expression in mpkCCD cells. (**a**) Tolvaptan promotes Nrf2 nuclear translocation. (Left panel) Western blotting of Nrf2 in nuclear extract. mpkCCD cells were treated with 200 μM tolvaptan or 5 μM sulforaphane on filter for 12 h, following which nuclear fraction was separated using commercially available reagents for nuclear extraction. Arrow indicates the band of Nrf2. (Right panel) Densitometric analysis of Nrf2. Error bars are mean values ± S.E. from three experiments. Tukey’s test, *P < 0.05. C: control (DMSO), Tol: 200 μM tolvaptan, Sul: 5 μM sulforaphane. P.C.: positive control. (**b**) Tolvaptan increases HO-1 mRNA expression in a dose-dependent manner. mpkCCD cells were treated with 10–200 μM tolvaptan for 4 h, following which HO-1 mRNA expression was examined using qPCR. Error bars are mean values ± S.E. from three experiments. Tukey’s test, *P < 0.05, **P < 0.01. (**c**) Tolvaptan induces HO-1 protein expression in a dose-dependent manner. (Top panel) mpkCCD cells were treated with 10–200 μM tolvaptan for 12 h. (Bottom panel) Densitometric analysis of HO-1 is presented. Error bars are mean values ± S.E. from three experiments. Tukey’s test, *P < 0.05, **P < 0.01. (**d**) An Nrf2 inhibitor, ML385, inhibits the HO-1 induction by tolvaptan. (Top panel) Following the pre-treatment of mpkCCD cells using DMSO (C, T, and S) or 50 μM ML385 (T + M and S + M) for 1 h, mpkCCD cells were treated with 200 μM tolvaptan or 5 μM sulforaphane in the presence or absence of 50 μM ML385 for 12 h. C: control (DMSO), T: 200 μM tolvaptan, M: 50 μM ML385, S: 5 μM sulforaphane. (Bottom panel) Densitometric analysis of HO-1 is presented. Error bars are mean values ± S.E. from three experiments. Tukey’s test, *P < 0.05, **P < 0.01.

To evaluate the effect of tolvaptan on Nrf2 signaling in the kidney, we administered tolvaptan to mpkCCD cells and collected the nuclear extract to evaluate Nrf2 nuclear translocation. The effect of tolvaptan was compared with that of known Nrf2 activator, sulforaphane, as a positive control. Nrf2 nuclear translocation was significantly increased by tolvaptan in mpkCCD cells (Fig. 1a). Further, tolvaptan increased HO-1 mRNA and protein expression (Fig. 1b,c). Tolvaptan-induced HO-1 expression was inhibited by an Nrf2 inhibitor, ML385 (Fig. 1d)^18^. These results indicated that tolvaptan promoted Nrf2 nuclear translocation and activated the antioxidant systems in mpkCCD cells.

### Tolvaptan activates the Nrf2/HO-1 antioxidant pathway through PERK phosphorylation

**Figure 2:**
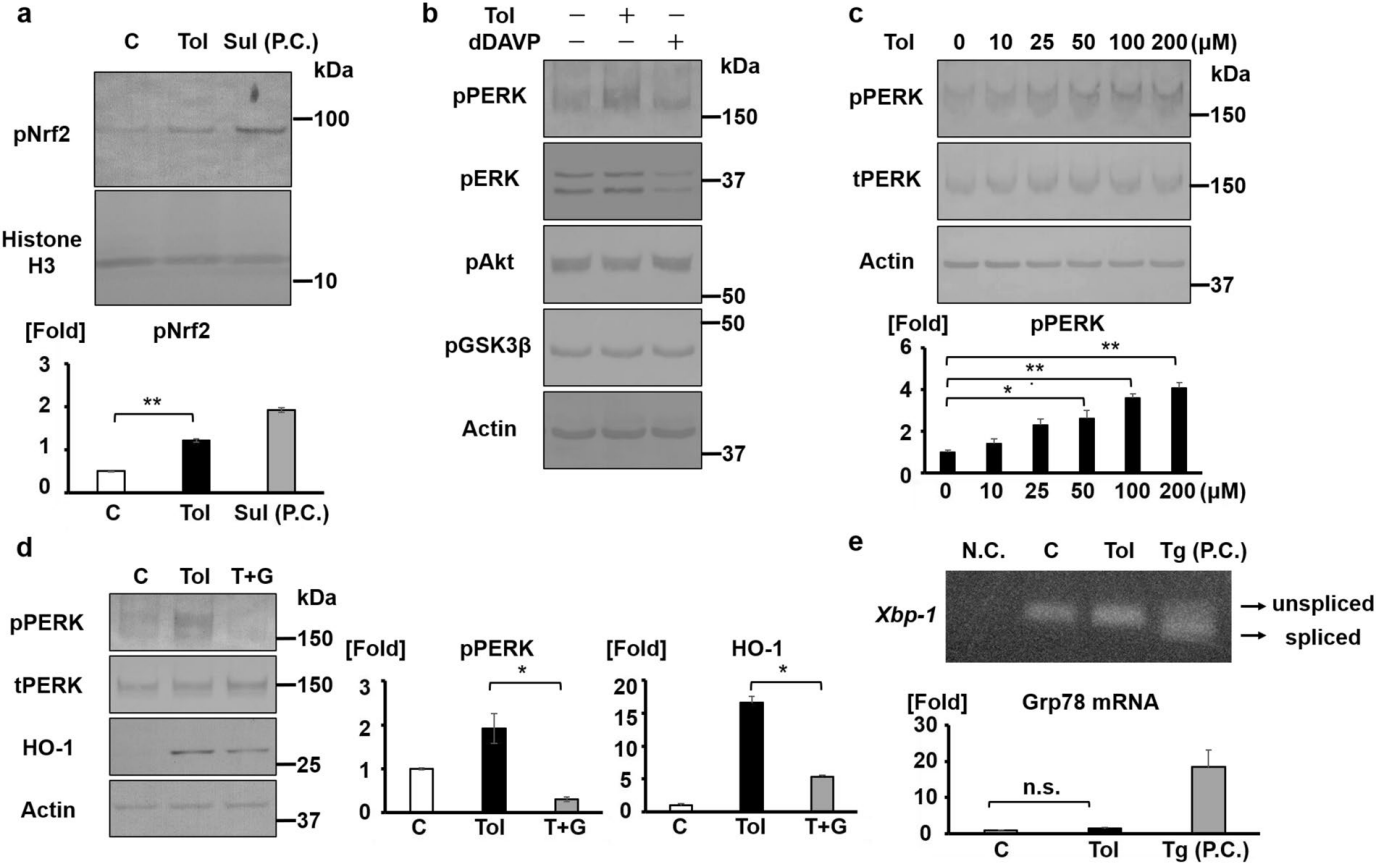
Tolvaptan activates the Nrf2/HO-1 antioxidant pathway through PERK phosphorylation. (**a**) Tolvaptan induces Nrf2 phosphorylation. (Top panel) mpkCCD cells were treated with 200 μM tolvaptan or 5 μM sulforaphane for 12 h, following which nuclear fraction was separated using commercially available reagents for nuclear extraction. (Bottom panel) pNrf2 bands were quantified by densitometric analysis. Error bars are mean values ± S.E. from three experiments. Tukey’s test, **P < 0.01. C: control (DMSO), Tol: 200 μM tolvaptan, Sul: 5 μM sulforaphane. P.C.: positive control. (**b**) Tolvaptan activates only PERK among upstream kinases of Nrf2. mpkCCD cells were treated with 200 μM tolvaptan or 1 nM dDAVP for 12 h. (**c**) Tolvaptan induces PERK phosphorylation in a dose-dependent manner. (Top panel) Tolvaptan (10–200 μM) was added to the basolateral side of mpkCCD cells for 12 h. (Bottom panel) Phospho-PERK bands were quantified by densitometric analysis. Error bars are mean values ± S.E. from three experiments. Tukey’s test, *P < 0.05, **P < 0.01. (**d**) A PERK inhibitor, GSK2606414, counteracts the effect of tolvaptan in inducing HO-1. (Left panel) Following the pre-treatment of mpkCCD cells using DMSO (C and Tol) or 5 μM GSK2606414 (T + G), 200 μM tolvaptan was added in the presence or absence of 5 μM GSK2606414. (Right panel) Densitometric analysis of pPERK and HO-1. Error bars are mean values ± S.E. from three experiments. Tukey’s test, *P < 0.05. C: control (DMSO), Tol: 200 μM tolvaptan, T + G: 200 μM tolvaptan and 5 μM GSK2606414. (**e**) Tolvaptan does not induce the IRE-1/XBP-1 pathway. (Top panel) mpkCCD cells were treated with 200 μM tolvaptan or 1 mM thapsigargin for 4 h. RT–PCR analysis of unspliced and spliced *Xbp-1* mRNA were performed. Representative data of three independent experiments are shown. (Bottom panel) The mRNA expression of *Grp78* is not increased by tolvaptan. The mRNA expression of *Grp78* was examined using qPCR. Error bars are mean values ± S.E. from three experiments. Tukey’s test, *P* < 0.05. C: control (DMSO), Tol: 200 μM tolvaptan, Tg: 1 μM thapshigarin. N.C.: negative control using water as the template, P.C.: positive control, n.s.: not significant.

Further, we investigated the key mediators of the tolvaptan/Nrf2/HO-1 signaling pathway. In previous reports, Nrf2 phosphorylation as well as oxidative stress disrupted Keap1–Nrf2 interaction and activated the antioxidant systems^10^. Nrf2 is directly phosphorylated by protein kinase RNA-like endoplasmic reticulum kinase (PERK) and is indirectly phosphorylated by extracellular signaling-regulated kinase (ERK), protein kinase B (Akt), and glycogen synthase kinase 3β (GSK3β)^19–22^. We examined the activities of these kinases using their phospho-specific antibodies because tolvaptan significantly increased Nrf2 phosphorylation (Fig. 2a)^22–25^. Tolvaptan phosphorylated only PERK in a dose-dependent manner in mpkCCD cells (Fig. 2b,c). We further confirmed that a PERK inhibitor, GSK2606414, significantly attenuated the effect of tolvaptan on HO-1 protein expression (Fig. 2d). These results indicated that PERK was an important mediator of tolvaptan-induced Nrf2/HO-1 activation. However, HO-1 induction was not sufficiently inhibited by GSK2606414 despite complete dephosphorylation of PERK (Fig. 2d), suggesting that other intracellular signaling molecules also mediated tolvaptan-induced HO-1 expression.

PERK is known as the sensor of endoplasmic reticulum (ER) stress, and it is phosphorylated in response to accumulation of ER stress^26^. In addition to PERK, ER stress phosphorylates another ER stress sensor, inositol-requiring enzyme 1 (IRE-1), which activates the splicing of *X-box-binding protein-1* (*Xbp-1*) mRNA and increases the expression of the ER chaperon, glucose-regulated protein 78 (Grp78)^26^. Therefore, we examined whether tolvaptan could simultaneously activate the IRE-1 signaling pathway. The effect of tolvaptan was compared with that of ER stress inducer, thapsigargin, as a positive control. Although thapsigargin induced the splicing of *Xbp-1* mRNA and increased the expression of *Grp78* mRNA, tolvaptan did not affect the IRE-1/XBP-1 pathway (Fig. 2e). These results indicated that tolvaptan could selectively activate the PERK signaling pathway without activation of IRE-1.

### Tolvaptan induces Nrf2 nuclear translocation and HO-1 expression *in vivo*

**Figure 3:**
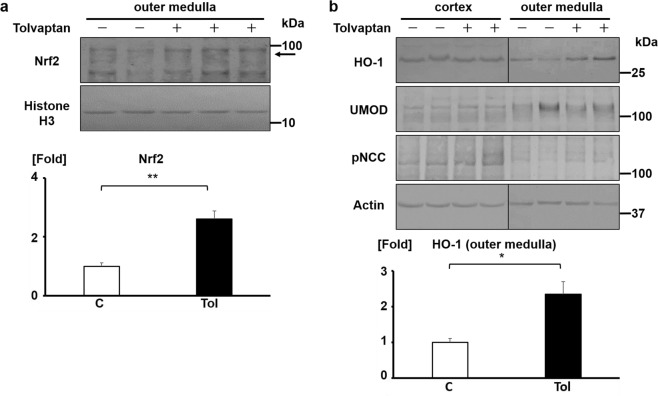
Tolvaptan induces Nrf2 nuclear translocation and HO-1 expression *in vivo*. (**a**) Tolvaptan promotes Nrf2 nuclear translocation in the outer medulla of mouse kidneys. (Top panel) Male C57BL/6J mice aged 8–9-weeks were fed with a diet containing 0.5% tolvaptan for 24 h. The nuclear extract was obtained from the outer medulla. Arrow indicates the band of Nrf2. (Bottom panel) Nrf2 bands were quantified using densitometric analysis. Bar indicates average from four or five experiments (control: n = 4, tolvaptan: n = 5). Student’s *t*-test, **P < 0.01. C: control, Tol: 0.5% tolvaptan. (**b**) Tolvaptan increases HO-1 protein expression in the outer medulla of mouse kidneys. (Top panel) Tolvaptan (0.5%) was administered via diet to 8–9-week-old male C57BL/6J mice for 24 h. The kidneys were separated into the cortex and the outer medulla, and the whole protein extraction was performed. The blots of HO-1 and Actin were configured with the blot of cortex and that of outer medulla. Fractionation of the cortex and the outer medulla was verified using UMOD and pNCC antibodies. (Bottom panel) Densitometric analysis of HO-1 is shown. Bar indicates average from four or five experiments (control: n = 4, tolvaptan: n = 5). Student’s *t*-test, *P < 0.05. C: control, Tol: 0.5% tolvaptan.

We verified the effect of tolvaptan on the Nrf2/HO-1 antioxidant pathway *in vivo*; to this end, we administered 0.5% tolvaptan via diet to male C57BL/6J mice for 24 h because 0.05%–0.5% tolvaptan in diet is required to attenuate renal damage^6,7^. Further, we measured the amount of water and feed intake and calculated the total 24 h dose of tolvaptan. Mean water intake was 10.5 ± 1.7 g in the control group and 35.6 ± 5.3 g in the tolvaptan-treated group. The average dose of tolvaptan was 23.8 ± 7.7 mg. On evaluating Nrf2 nuclear translocation and HO-1 protein expression in the kidneys using nuclear extracts (Table 1) and whole tissue lysates (Table 2) of tolvaptan-treated mice, it was observed that although tolvaptan did not increase HO-1 protein expression in the renal cortex, it could successfully induce Nrf2 nuclear translocation and HO-1 protein expression in the renal outer medulla (Fig. 3a,b). However, no PERK phosphorylation was detected by western blotting and immunostaining analysis using commercially available antibodies.

**Table 1:** The amount of water intake and the dose of tolvaptan of mice analyzed as detailed in Fig. 3a.

	Water intake (g)	Dose of tolvaptan (mg)
Control 1	10.9	0
Control 2	12.8	0
Control 3	10.6	0
Control 4	10.0	0
Tolvaptan 1	36.2	31
Tolvaptan 2	33.7	44
Tolvaptan 3	39.0	20.5
Tolvaptan 4	45.0	22.0
Tolvaptan 5	42.3	25.0

The dose of tolvaptan was calculated from the amount of feed intake. The average amount of water intake is 11.1 ± 1.0 g in control group and 39.2 ± 4.1 g in the tolvaptan-treated group. The average dose of tolvaptan is 28.5 ± 8.5 mg.

**Table 2:** The amount of water intake and the dose of tolvaptan of mice analyzed as detailed in Fig. 3b.

	Water intake (g)	Dose of tolvaptan (mg)
Control 1	7.4	0
Control 2	9.1	0
Control 3	12.9	0
Control 4	10.4	0
Tolvaptan 1	27.1	20.5
Tolvaptan 2	28.9	21.5
Tolvaptan 3	37.0	19.0
Tolvaptan 4	33.6	17.5
Tolvaptan 5	33.1	17.0

The dose of tolvaptan was calculated from the amount of feed intake. The average amount of water intake is 9.95 ± 2.0 g in control group and 31.9 ± 3.5 g in the tolvaptan-treated group. The average dose of tolvaptan is 19.1 ± 1.7 mg.

### Tolvaptan activates the Nrf2/HO-1 antioxidant pathway independently of cAMP signaling

**Figure 4:**
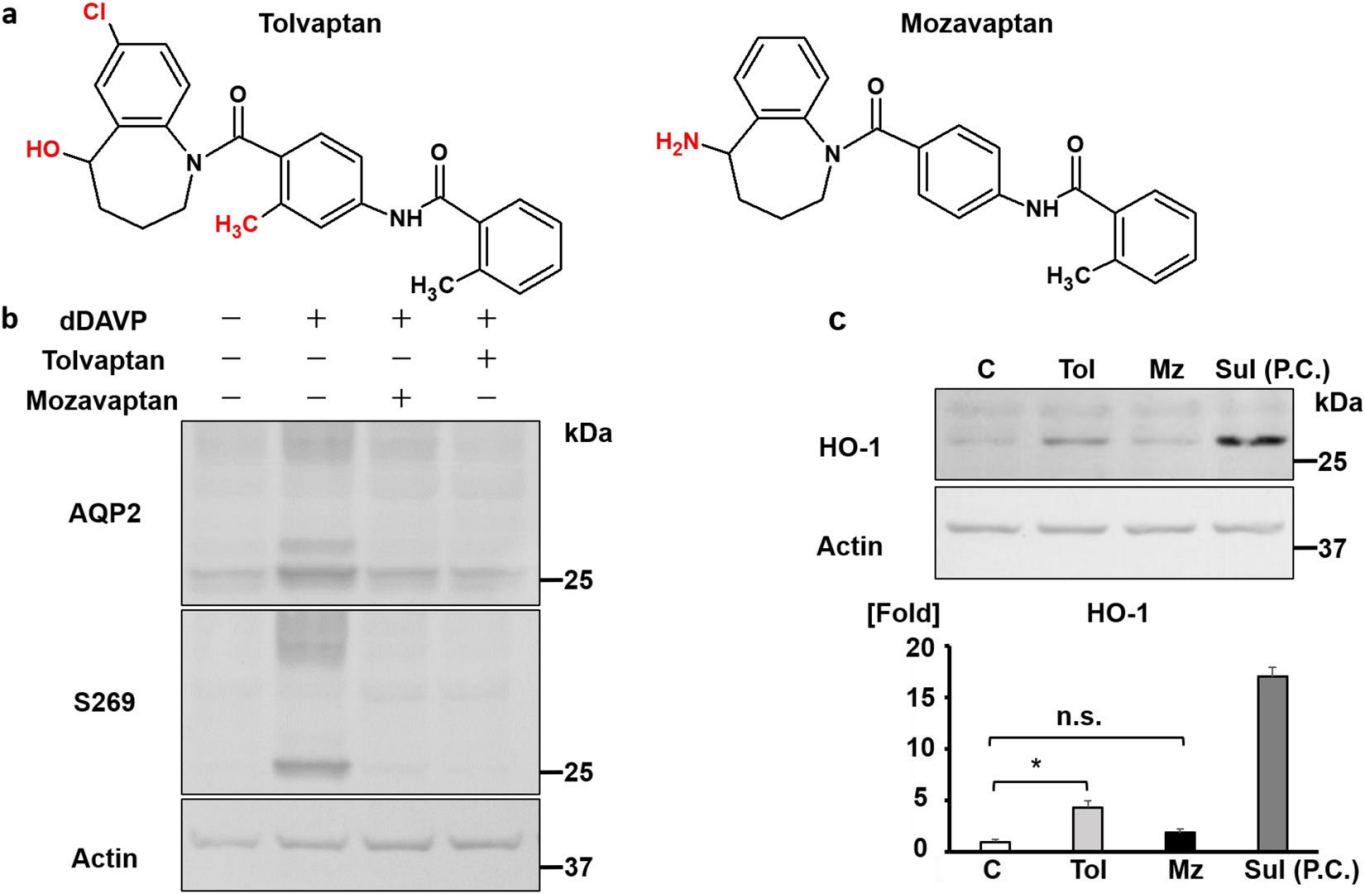
Tolvaptan activates the Nrf2/HO-1 antioxidant pathway independently of cAMP signaling. (**a**) The chemical structure of mozavaptan is similar to that of tolvaptan. The structural difference between tolvaptan and mozavaptan is indicated in red. (**b**) Both tolvaptan and mozavaptan completely counteracts the effect of dDAVP on AQP2 phosphorylation at S269. Following the pre-incubation of mpkCCD cells using DMSO, 200 μM tolvaptan, or 100 μM mozavaptan for 1 h, 1 nM dDAVP was added for 12 h. The representative blots of three independent experiments are shown. (**c**) Mozavaptan does not induce HO-1 in mpkCCD cells. (Top panel) mpkCCD cells were treated with 200 μM tolvaptan or 100 μM mozavaptan for 12 h. (Bottom panel) Densitometric analysis of HO-1 is presented. Error bars are mean values ± S.E. from three experiments. Tukey’s test, *P < 0.05. C: control (DMSO), Tol: 200 μM tolvaptan, Mz: 100 μM mozavaptan, Sul: 5 μM sulforaphane. P.C.: positive control, n.s.: not significant.

Considering that tolvaptan activates the Nrf2/HO-1 pathway, other V2R antagonists could probably exert the same effect. Therefore, we examined the effect of mozavaptan, which has a chemical structure quite similar to that of tolvaptan (Fig. 4a). The administered doses of tolvaptan and mozavaptan were sufficiently high to inhibit vasopressin/cAMP signaling and completely counteracted the effect of [deamino-Cys1, d-Arg8]-vasopressin (dDAVP) on aquaporin-2 (AQP2) phosphorylation at serine 269 (Fig. 4b)^5,27^. Nevertheless, in contrast to tolvaptan, mozavaptan did not induce HO-1 protein expression in mpkCCD cells (Fig. 4c). These results indicate that the suppression of cAMP signaling is insufficient to induce HO-1 transcription.

### Tolvaptan and BARD synergistically activate the Nrf2/HO-1 antioxidant pathway

**Figure 5:**
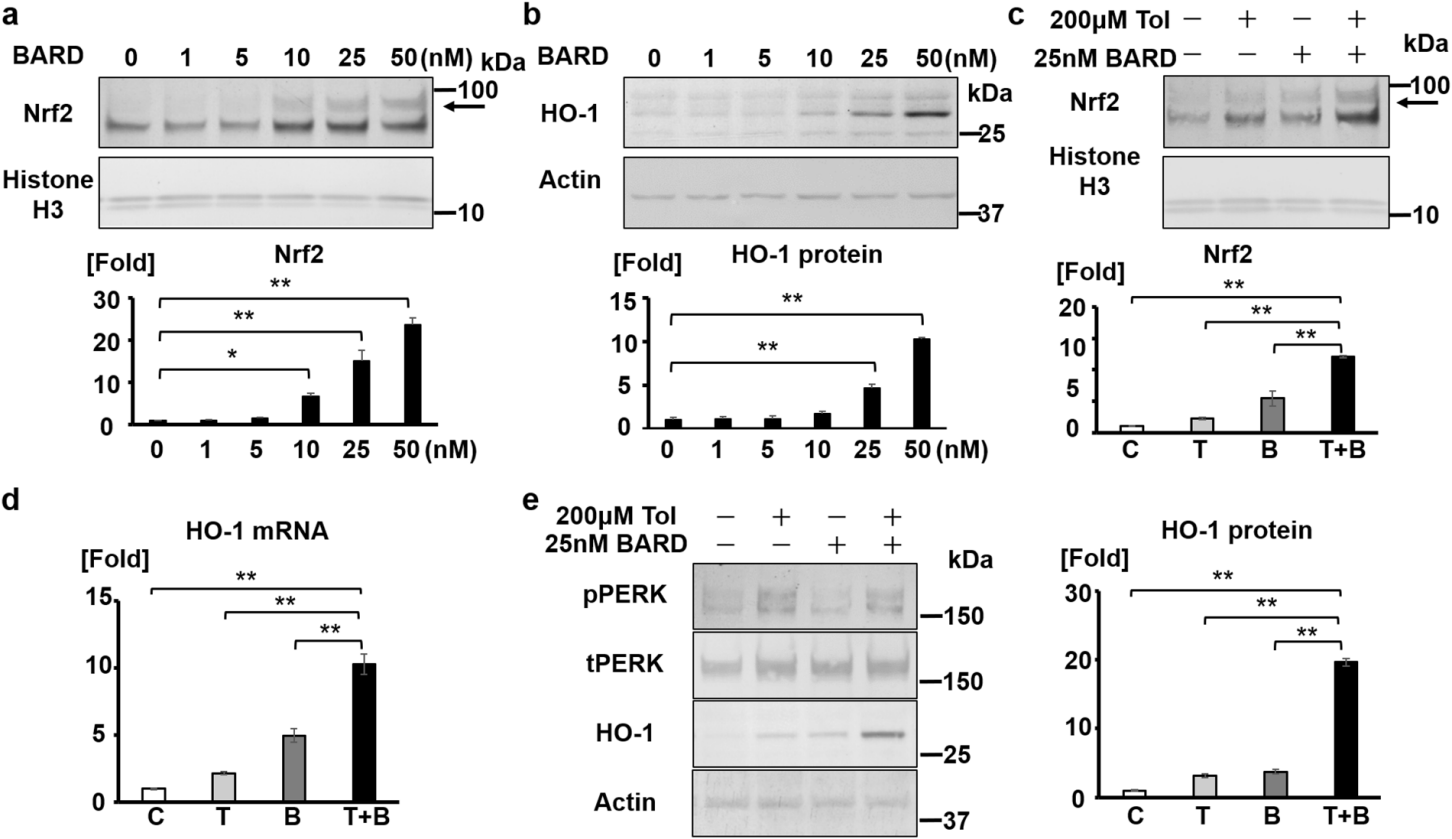
Tolvaptan and BARD synergistically activates the Nrf2/HO-1 antioxidant pathway. (**a**) BARD promotes Nrf2 nuclear translocation in a dose-dependent manner. (Top panel) mpkCCD cells were treated with 1–50 nM BARD for 12 h. The nuclear extract was obtained using nuclear extraction reagents to evaluate Nrf2 nuclear translocation. Arrow indicates the band of Nrf2. (Bottom panel) Densitometric analysis of Nrf2 is presented in the bar graphs. Error bars are mean values ± S.E. from three experiments. Tukey’s test, *P < 0.05, **P < 0.01. (**b**) BARD increases HO-1 protein expression. (Top panel) mpkCCD cells were treated with 1–50 nM BARD for 12 h. (Bottom panel) Densitometric analysis of HO-1 is presented. Error bars are mean values ± S.E. from three experiments. Tukey’s test, **P < 0.01. (**c**) Tolvaptan and BARD synergistically increases Nrf2 nuclear translocation. (Top panel) Tolvaptan (200 μM) was administered to mpkCCD cells in the presence or absence of BARD (25 nM) for 12 h. (Bottom panel) Densitometric analysis of Nrf2 is presented. Error bars are mean values ± S.E. from three experiments. Tukey’s test, **P < 0.01. C: control (DMSO), T: 200 μM tolvaptan, B: 25 nM BARD, T + B: 200 μM tolvaptan and 25 nM BARD. (**d**) Tolvaptan and BARD synergistically increases HO-1 mRNA expression. Tolvaptan (200 μM) was administered to mpkCCD cells in the presence or absence of BARD (25 nM) for 4 h. HO-1 mRNA expression was examined using qPCR. Error bars are mean values ± S.E. from three experiments. Tukey’s test, **P < 0.01. C: control (DMSO), T: 50 μM tolvaptan, B: 25 nM BARD, T + B: 200 μM tolvaptan and 25 nM BARD. (**e**) Tolvaptan and BARD synergistically increases HO-1 protein expression. (Top panel) The representative western blotting of HO-1. (Bottom panel) Densitometric analysis of HO-1 is presented. Error bars are mean values ± S.E. from three experiments. Tukey’s test, **P < 0.01. C: control (DMSO), T: 200 μM tolvaptan, B: 25 nM BARD, T + B: 200 μM tolvaptan and 25 nM BARD.

Tolvaptan activated the Nrf2/HO-1 pathway via a different mechanism to that of BARD. BARD interacts with cysteine residues of Keap1 and inhibits the Keap1–Nrf2 binding, leading to Nrf2 nuclear translocation^28^. In contrast, tolvaptan activated Nrf2 through PERK phosphorylation. We examined whether tolvaptan and BARD additively or synergistically activate the Nrf2/HO-1 antioxidant pathway and found that BARD of concentration >25 nM activated the Nrf2/HO-1 antioxidant pathway in mpkCCD cells (Fig. 5a,b). Interestingly, 200 μM tolvaptan plus 25 nM BARD synergistically activated Nrf2 nuclear translocation and increased HO-1 mRNA and protein expression (Fig. 5c–e). PERK phosphorylation was only induced by tolvaptan.

## Discussion

**Figure 6:**
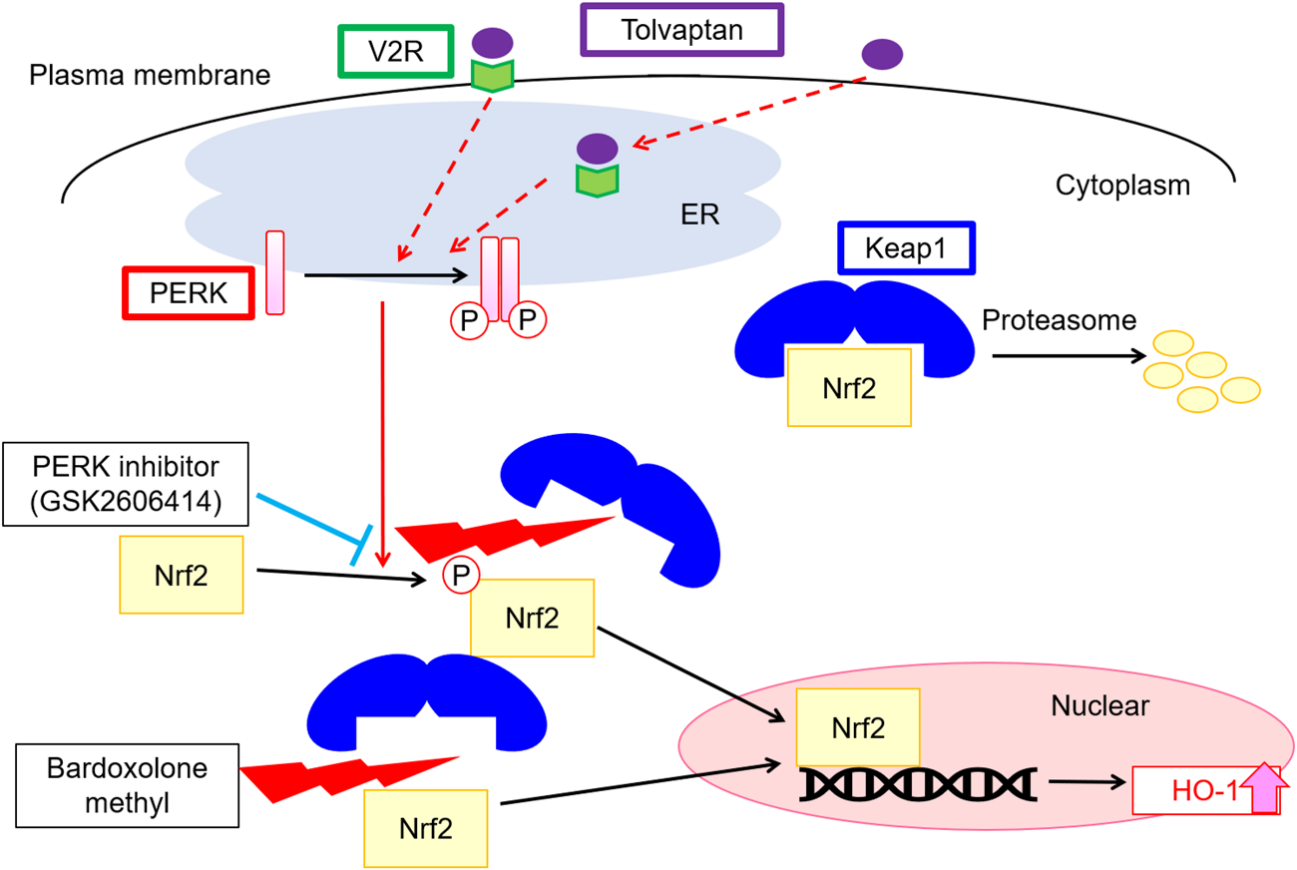
The schematic summary of the regulation of the Nrf2/HO-1 antioxidant pathway by tolvaptan. Tolvaptan phosphorylated PERK independently of cAMP signaling, following which PERK directly phosphorylates Nrf2, leading to the dissociation of Nrf2 from Keap1^10,25^. Once released from Keap1, Nrf2 is not degraded by proteasome and translocates from the cytosol to nucleus. Tolvaptan increases the transcription of an antioxidant enzyme HO-1 in mpkCCD cells and the outer medulla of mouse kidneys. A PERK inhibitor, GSK2606414, counteracted the effect of tolvaptan on HO-1 expression. Although the precise mechanisms remain unclear, tolvaptan may bind to membrane- and ER-localized V2R and modulate PERK phosphorylation. Tolvaptan activated Nrf2 via a different mechanism to that of BARD. As a consequence, the combination therapy of tolvaptan and BARD could synergistically activate the Nrf2/HO-1 antioxidant pathway.

In the present study, we clarified that tolvaptan activated the Nrf2/HO-1 antioxidant pathway in mpkCCD cells and the outer medulla of mouse kidneys. To date, the V2R antagonist tolvaptan has provided clinical benefits in patients with heart failure, SIADH, and ADPKD by inhibiting intracellular cAMP production in the kidney^1–4^. We found novel pharmacological properties of tolvaptan that upregulated the Nrf2-antioxidant systems independently of cAMP signaling (Fig. 4a–c). Although inflammation and oxidative stress are prevalent in CKD, paradoxical Nrf2-dysregulation and unresponsiveness of antioxidant enzymes are observed^13,29,30^. To improve this situation, Nrf2 activators, such as BARD, have received much attention as the next-generation therapeutic target of CKD. Therefore, the activation of tolvaptan/PERK/Nrf2/HO-1 signaling pathway is a potential therapeutic target of CKD (Fig. 6).

Moreover, we found that tolvaptan and BARD synergistically activated the Nrf2/HO-1 antioxidant pathway (Fig. 5c–e). In addition to the enhancement of their drug efficacies, tolvaptan offers the possibility of avoiding the undesirable side-effects of BARD. BARD increased cardiovascular diseases, particularly heart failure, in the phase-3 Bardoxolone Methyl Evaluation in Patients with Chronic Kidney Disease and Type 2 Diabetes Mellitus: the Occurrence of Renal Events (BEACON) trial, resulting in premature termination of the trial^31^. In the BARD-treated group, fluid retention may have caused hemodilution, thereby increasing blood pressure, and leading to a higher incidence of heart failure. On the contrary, the diuretic effect of tolvaptan decreases fluid overload without deterioration of the renal functions in patients with heart failure and CKD^32^. Consequently, the combined therapy of tolvaptan and BARD may improve BARD-induced positive fluid balance and contribute to the treatment of CKD via synergistic induction of the antioxidant defense systems.

Tolvaptan-induced Nrf2/HO-1 antioxidant pathway was mediated by ER-localized transmembrane kinase, PERK (Fig. 2b–d); however, the precise mechanism of PERK phosphorylation remains unclear. Interestingly, tolvaptan is known as a cell-permeable pharmacological chaperon that can directly bind to misfolded V2R mutants retained in the ER and thereby facilitate their proper folding and plasma membrane trafficking^33,34^. Both V2R mutants and wild-type V2R are constitutively misfolded and degraded in the ER. Protein folding efficiency of G-protein coupled receptors, including V2R, is only <50%^35^. Tolvaptan may directly bind to misfolded wild-type V2R in the ER and subsequently modulate PERK phosphorylation without activation of the ER stress transducer, IRE-1 (Figs 2e, 6). The significant difference in terms of HO-1 activation between tolvaptan and mozavaptan further supports our notion (Fig. 4c). Tolvaptan is a more potent pharmacological chaperon than mozavaptan, and only tolvaptan has been shown to increase membrane trafficking of wild-type V2R^34^. The ability of tolvaptan to bind to wild-type V2R in the ER suggests that it can activate the PERK/Nrf2/HO-1 signaling pathway in other V2R-expressing cells. Indeed, tolvaptan activated Nrf2-regulated antioxidant enzymes, such as HO-1 and NAD(P)H:quinone oxidoreductase-1 (NQO-1), in heart-derived H9C2 cells, which endogenously expressed V2R (Supplementary Fig. 1a–c)^11,36^. Tolvaptan may activate the antioxidant systems in extra-renal organs as well as in kidneys. Conversely, tolvaptan was not effective in renal proximal tubule-derived HK2 cells, which did not endogenously express V2R (Supplementary Fig. 2a–c)^37^.

The Nrf2/HO-1 antioxidant pathway was successfully activated in the renal outer medulla of tolvaptan-treated mice. Mean dose of tolvaptan for mice in the present study was approximately 1000 mg/kg/day (Tables 1 and 2). Based on the results of the area under the curve of tolvaptan, the exposure level of 1000 mg/kg/day tolvaptan in a mouse is equivalent to that of 250 mg/day in a human^38^. In a previous report, 0.05% tolvaptan in diet improved the renal function and histopathology in a rodent model of end-stage heart failure^7^. Moreover, 0.1% tolvaptan is protective against podocyte damage and proteinuria in a rodent model of puromycin aminonucleoside induced-nephrosis^6^. These beneficial effects of tolvaptan are presumably partly caused by the activation of the Nrf2/HO-1 antioxidant pathway. Previous evidence and our results propose that 25–250 mg/day tolvaptan in clinical use may exert renal protective effects. In addition, the combination therapy of tolvaptan and BARD is a promising strategy to reduce the dose of tolvaptan.

In the kidney, V2R is strongly expressed in the outer medulla^37^. In addition, tolvaptan antagonizes the binding of vasopressin to V2R especially in the medulla region of the kidney^39^. Based on the fact that tolvaptan induced antioxidant pathway in the outer medulla (Fig. 3b), tolvaptan may contribute to the treatment of acute kidney injury (AKI) as well as CKD. Due to the relatively larger consumption of O_2_, the renal medulla is vulnerable to hypoxia. Nrf2 activation is a promising therapeutic target to ameliorate tubular necrosis and apoptosis of the outer medulla caused by ischemic renal injury^40^. Tolvaptan is a potential therapeutic target of ischemic AKI which is a common clinical event and causes progression of CKD.

In conclusion, we found the novel pharmacological property of tolvaptan that activated the PERK/Nrf2/HO-1 signaling pathway. Nrf2-regulated antioxidant systems were synergistically activated by tolvaptan and BARD. Tolvaptan is a potential therapeutic candidate in renal disease.

## Methods

### Cell cultures

mpkCCD cells (a contribution from Alain Vandewalle, Paris) were cultured in modified DM medium as previously described^41,42^ and were seeded on semipermeable filters (Transwell 0.4-μm pore size, 4.67 cm2; Corning Costar). The cells were cultured for 5 days, following which they were serum-starved and hormone-deprived for 12 h. The culture medium was changed daily. Tolvaptan (LKT Laboratories) (10–200 μM), L-sulforaphane (Sigma-Aldrich) (10 μM), ML385 (Selleck) (50 μM), dDAVP (Sigma-Aldrich) (1 nM), GSK2606414 (Sigma-Aldrich) (5 μM), thapsigargin (Sigma-Aldrich) (1 μM), and bardoxolone methyl (Cayman Chemical) (1–50 nM), and mozavaptan (Cayman Chemical) (100 μM) were applied to the basolateral side of the mpkCCD cells. The H9C2 cells were cultured in DMEM (Nacalai Tesque) supplemented with 10% fetal calf serum, 2 mM L-glutamine, 100 U/mL penicillin, and 0.1 mg/mL streptomycin. On reaching 70–80% confluence, cells were exposed to tolvaptan (200 μM), L-sulforaphane (10 μM), and dDAVP (Sigma-Aldrich) (1 nM). The renal proximal tubule-derived HK-2 cells were cultured in modified DM medium with 10% fetal calf serum, 100 U/ml penicillin, and 0.1 mg/mL streptomycin. HK2 cells were treated with tolvaptan (200 μM), L-sulforaphane (10 μM), and dDAVP (Sigma-Aldrich) (1 nM) at 90–95% confluence. All reagents were solved with dimethyl sulfoxide (DMSO).

### Animals

All experiments were performed in accordance with the guidelines for animal research of Tokyo Medical and Dental University, and the study protocol was approved by The Animal Care and Use Committee of Tokyo Medical and Dental University (approval number: A2019-183C3). Male C57BL/6J mice (8–9-week-old) (CLEA JAPAN) were maintained under standard lightning conditions (12 h:12 h light-dark cycle). The mice were randomly divided into two groups: the control group (n = 8) and the tolvaptan-treated group (n = 10). Individual housing (one per cage) was provided for all mice, and they had free access to water and feed. The control group received a normal chow without tolvaptan, whereas the tolvaptan-treated group received a normal chow with 0.5% tolvaptan for 24 h. Following the administration of tolvaptan, the mice were euthanized and their kidneys were removed and separated into the cortex and outer medulla for protein extraction.

### Western blotting

Whole homogenates of mouse cortex and outer medulla without the nuclear fraction (600 × *g*) were prepared as previously described^43^. mpkCCD cells were solubilized in lysis buffer as previously described^42^. H9C2 cells were lysed in RIPA-P buffer (50 mM Tris-HCl; pH 7.5, 150 mM NaCl, 0.5% sodium deoxycholate, 0.1% SDS, 1 mM EGTA, 1 mM EDTA, 1 mM sodium orthovanadate, 50 mM sodium fluoride, 1% Triton X-100, and protease inhibitor cocktail; Roche Diagnostics). Both the cells were lysed for 30 min at 4 °C. The cell lysate was centrifuged at 12,000 × *g* for 10 min at 4 °C, following which supernatants were diluted with 2 × SDS sample buffer (Cosmo Bio) and denatured at 60 °C for 20 min. The nuclear extract was used to measure the levels of Nrf2 nuclear translocation and phosphorylation. The nuclear extraction from mouse outer medulla, mpkCCD cells, and H9C2 cells using NE-PER nuclear and cytoplasmic extraction reagents (ThermoFisher Scientific) was performed according to the manufacturer’s instructions. Equal amounts of protein were separated by SDS-PAGE and were transferred onto nitrocellulose membrane (GE Healthcare Life Sciences). Fractionation of the cortex and the outer medulla was verified using uromodulin (UMOD) and phospho-sodium-chloride transporter (pNCC) antibodies. The blots were probed with the following primary antibodies: rabbit anti-HO-1 (Enzo Life Sciences, ADI-SPA-895-F; 1:1000), mouse anti-HO-1 (Abcam, ab13248), mouse anti-NQO-1 (Abcam, ab28947, 1:1000), rabbit anti-Nrf2 (Cell Signaling, #12721; 1:1,000), rabbit anti-phospho-Nrf2 (Abcam, ab76026, 1:1000), rabbit anti-PERK (Cell Signaling, #3192; 1:1000), rabbit anti-phospho-PERK (Thr 980) (Cell Signaling, # 3179; 1:1,000), rat anti-UMOD (R&D systems, MAB5175; 1:1,000), rabbit anti-phospho-NCC (S71)^43^, rabbit anti-Histone H3 (Cell Signaling, #4499; 1:1,000), goat anti-AQP2 (N-20, Santa Cruz, sc-9880; 1:1,000), rabbit anti-phospho-AQP2 (S269) (Symansis, p112-269; 1:1,000), rabbit anti-phospho-p44/42 MAPK (Thr202/Tyr204) (Cell Signaling, #9101; 1:1,000), rabbit anti-phospho-Akt (Ser 473) (Cell Signaling, #4060; 1:1,000), rabbit anti-phospho-GSK-3β (Ser9) (Cell Signaling, #9322; 1:1,000), and rabbit anti-actin (Cytoskeleton, #AAN01; 1:1,000). Alkaline phosphatase-conjugated anti-rabbit IgG antibody (Promega), anti-goat IgG antibody (Promega), and anti-rat IgG antibody (Abcam) were used as secondary antibodies. The band intensities of the western blots were quantified using ImageJ software.

### Reverse transcription–polymerase chain reaction (RT–PCR) analysis

Total RNA was extracted using the Sepazol®-RNA ISuper G (Nacarai Tesque), and cDNA was synthesized using the ReverTra® Ace (Toyobo), according to the manufacturer’s instruction. The forward and reverse *Xbp-1* primers used were the same as previously described^44^. PCR amplification consisted of 35 cycles (95 °C for 10 s, 62 °C for 15 s, 72 °C for 30 s) after an initial denaturation step at 95 °C for 3 min. The PCR products were analyzed by electrophoresis on 2.0% agarose gel.

### Quantitative real-time PCR (qPCR) analysis

Total RNA was extracted using the Sepazol®-RNA ISuper G (Nacarai Tesque), and cDNA was synthesized using the ReverTra® Ace (Toyobo). qPCR analysis was performed in the Thermal Cycler Dice Real Time System (Takara Bio). Primers and templates were mixed using SYBR Premix Ex Taq II (Takara Bio). All reactions were performed in triplicates. The transcript levels were normalized to the GAPDH mRNA levels, and the amount of RNA was calculated using the comparative C_T_ method. The forward and reverse primers used for the detection of mouse *Ho-1* were 5ʹ-CGCCTTCCTGCTCAACATT-3ʹ and 5ʹ-TGTGTTCCTCTGTCAGCATCAC-3ʹ respectively. The forward and reverse primers used for the detection of mouse *Grp78* were 5ʹ-ATATTGGAGGTGGGCAAAC-3ʹ and 5ʹ-CAT CTTTGGTTGCTTGTCG-3ʹ, respectively.

### Statistics

Statistical significance was evaluated using one-way ANOVA test with multiple comparisons using Tukey’s correction. Data are presented as means ± S.E. In the analysis of *in vivo* experiments, unpaired Student’s *t*-tests were performed to assess the statistical significance. *P* < 0.05 was considered statistically significant.

## Supplementary information

Supplementary Figure

## Data Availability

All data are available from the corresponding author upon reasonable request.

## References

[1] Gheorghiade M (2004). Effects of tolvaptan, a vasopressin antagonist, in patients hospitalized with worsening heart failure: a randomized controlled trial. JAMA.

[2] Schrier RW (2006). Tolvaptan, a selective oral vasopressin V2-receptor antagonist, for hyponatremia. N Engl J Med.

[3] Torres VE (2012). Tolvaptan in patients with autosomal dominant polycystic kidney disease. N Engl J Med.

[4] Torres VE, Harris PC (2014). Strategies targeting cAMP signaling in the treatment of polycystic kidney disease. J Am Soc Nephrol.

[5] Ando F, Uchida S (2018). Activation of AQP2 water channels without vasopressin: therapeutic strategies for congenital nephrogenic diabetes insipidus. Clin Exp Nephrol.

[6] Okada T (2009). Tolvaptan, a selective oral vasopressin V2 receptor antagonist, ameliorates podocyte injury in puromycin aminonucleoside nephrotic rats. Clin Exp Nephrol.

[7] Ishikawa M, Kobayashi N, Sugiyama F, Onoda S, Ishimitsu T (2013). Renoprotective effect of vasopressin v2 receptor antagonist tolvaptan in Dahl rats with end-stage heart failure. Int Heart J.

[8] Stenvinkel P (2018). Novel treatment strategies for chronic kidney disease: insights from the animal kingdom. Nat Rev Nephrol.

[9] Itoh K (1999). Keap1 represses nuclear activation of antioxidant responsive elements by Nrf2 through binding to the amino-terminal Neh2 domain. Genes Dev.

[10] Nguyen T, Sherratt PJ, Huang HC, Yang CS, Pickett CB (2003). Increased protein stability as a mechanism that enhances Nrf2-mediated transcriptional activation of the antioxidant response element. Degradation of Nrf2 by the 26 S proteasome. J Biol Chem.

[11] Ray PD, Huang BW, Tsuji Y (2012). Reactive oxygen species (ROS) homeostasis and redox regulation in cellular signaling. Cell Signal.

[12] Ricardo SD, Bertram JF, Ryan GB (1994). Reactive oxygen species in puromycin aminonucleoside nephrosis: *in vitro* studies. Kidney Int.

[13] Kim HJ, Vaziri ND (2010). Contribution of impaired Nrf2-Keap1 pathway to oxidative stress and inflammation in chronic renal failure. Am J Physiol Renal Physiol.

[14] Aminzadeh MA (2014). The synthetic triterpenoid RTA dh404 (CDDO-dhTFEA) restores Nrf2 activity and attenuates oxidative stress, inflammation, and fibrosis in rats with chronic kidney disease. Xenobiotica.

[15] Nezu M, Suzuki N, Yamamoto M (2017). Targeting the KEAP1-NRF2 System to Prevent Kidney Disease Progression. Am J Nephrol.

[16] Pergola PE (2011). Bardoxolone methyl and kidney function in CKD with type 2 diabetes. N Engl J Med.

[17] Chin MP (2018). Bardoxolone Methyl Improves Kidney Function in Patients with Chronic Kidney Disease Stage 4 and Type 2 Diabetes: Post-Hoc Analyses from Bardoxolone Methyl Evaluation in Patients with Chronic Kidney Disease and Type 2 Diabetes Study. Am J Nephrol.

[18] Singh A (2016). Small Molecule Inhibitor of NRF2 Selectively Intervenes Therapeutic Resistance in KEAP1-Deficient NSCLC Tumors. ACS Chem Biol.

[19] Cullinan SB (2003). Nrf2 is a direct PERK substrate and effector of PERK-dependent cell survival. Mol Cell Biol.

[20] Papaiahgari S, Kleeberger SR, Cho HY, Kalvakolanu DV, Reddy SP (2004). NADPH oxidase and ERK signaling regulates hyperoxia-induced Nrf2-ARE transcriptional response in pulmonary epithelial cells. J Biol Chem.

[21] Martin D (2004). Regulation of heme oxygenase-1 expression through the phosphatidylinositol 3-kinase/Akt pathway and the Nrf2 transcription factor in response to the antioxidant phytochemical carnosol. J Biol Chem.

[22] Jiang Y (2015). Therapeutic targeting of GSK3β enhances the Nrf2 antioxidant response and confers hepatic cytoprotection in hepatitis C. Gut.

[23] Harding HP, Zhang Y, Ron D (1999). Protein translation and folding are coupled by an endoplasmic-reticulum-resident kinase. Nature.

[24] Boulton TG (1991). ERKs: a family of protein-serine/threonine kinases that are activated and tyrosine phosphorylated in response to insulin and NGF. Cell.

[25] Sato S, Fujita N, Tsuruo T (2000). Modulation of Akt kinase activity by binding to Hsp90. Proc Natl Acad Sci USA.

[26] Kadowaki H, Nishitoh H (2013). Signaling pathways from the endoplasmic reticulum and their roles in disease. Genes (Basel).

[27] Ando F (2018). AKAPs-PKA disruptors increase AQP2 activity independently of vasopressin in a model of nephrogenic diabetes insipidus. Nat Commun.

[28] Dinkova-Kostova AT (2005). Extremely potent triterpenoid inducers of the phase 2 response: correlations of protection against oxidant and inflammatory stress. Proc Natl Acad Sci USA.

[29] Kim HJ, Sato T, Rodríguez-Iturbe B, Vaziri ND (2011). Role of intrarenal angiotensin system activation, oxidative stress, inflammation, and impaired nuclear factor-erythroid-2-related factor 2 activity in the progression of focal glomerulosclerosis. J Pharmacol Exp Ther.

[30] Aminzadeh MA, Nicholas SB, Norris KC, Vaziri ND (2013). Role of impaired Nrf2 activation in the pathogenesis of oxidative stress and inflammation in chronic tubulo-interstitial nephropathy. Nephrol Dial Transplant.

[31] de Zeeuw D (2013). Bardoxolone methyl in type 2 diabetes and stage 4 chronic kidney disease. N Engl J Med.

[32] Sen J, Chung E, McGill D (2018). Tolvaptan for Heart Failure in Chronic Kidney Disease Patients: A Systematic Review and Meta-Analysis. Heart Lung Circ.

[33] Bernier V (2004). Functional rescue of the constitutively internalized V2 vasopressin receptor mutant R137H by the pharmacological chaperone action of SR49059. Mol Endocrinol.

[34] Takahashi K (2012). V2 vasopressin receptor (V2R) mutations in partial nephrogenic diabetes insipidus highlight protean agonism of V2R antagonists. J Biol Chem.

[35] Beerepoot P, Nazari R, Salahpour A (2017). Pharmacological chaperone approaches for rescuing GPCR mutants: Current state, challenges, and screening strategies. Pharmacol Res.

[36] Kaufmann JE, Iezzi M, Vischer UM (2003). Desmopressin (DDAVP) induces NO production in human endothelial cells via V2 receptor- and cAMP-mediated signaling. J Thromb Haemost.

[37] Mutig K (2007). Vasopressin V2 receptor expression along rat, mouse, and human renal epithelia with focus on TAL. Am J Physiol Renal Physiol.

[38] Oi A (2011). Nonclinical safety profile of tolvaptan. Cardiovasc Drugs Ther.

[39] Serradeil-Le Gal C (1996). Characterization of SR 121463A, a highly potent and selective, orally active vasopressin V2 receptor antagonist. J Clin Invest.

[40] Nezu M (2017). Transcription factor Nrf2 hyperactivation in early-phase renal ischemia-reperfusion injury prevents tubular damage progression. Kidney Int.

[41] Bens M (1999). Corticosteroid-dependent sodium transport in a novel immortalized mouse collecting duct principal cell line. J Am Soc Nephrol.

[42] Ando F (2016). Wnt5a induces renal AQP2 expression by activating calcineurin signalling pathway. Nat Commun.

[43] Yang SS (2007). Molecular pathogenesis of pseudohypoaldosteronism type II: generation and analysis of a Wnk4(D561A/+) knockin mouse model. Cell Metab.

[44] Sha H (2009). The IRE1alpha-XBP1 pathway of the unfolded protein response is required for adipogenesis. Cell Metab.

