# Supplementary material for: Tolvaptan activates the Nrf2/HO-1 antioxidant pathway through PERK phosphorylation
Source: Sci Rep. 2019 Jun 25;9:9245. doi: 10.1038/s41598-019-45539-8 (PMC6592894; doi:10.1038/s41598-019-45539-8)

Tolvaptan activates the Nrf2/HO-1 antioxidant pathway through PERK phosphorylation

Tamami Fujiki, M.D.[1], Fumiaki Ando, M.D., Ph.D.[1], Kana Murakami[1], Kiyoshi Isobe, M.D., Ph.D.[1], Takayasu Mori, M.D., Ph.D.[1], Koichiro Susa, M.D., Ph.D.[1], Naohiro Nomura, M.D., Ph.D.[1], Eisei Sohara, M.D., Ph.D.[1], Tatemitsu Rai, M.D., Ph.D.[1], Shinichi Uchida, M.D., Ph.D.[1]

[1] Department of Nephrology, Tokyo Medical and Dental University (TMDU), Tokyo, Japan

Supplementary Figure 1.

Tolvaptan activates the Nrf2/HO-1 antioxidant pathway in H9C2 cells.

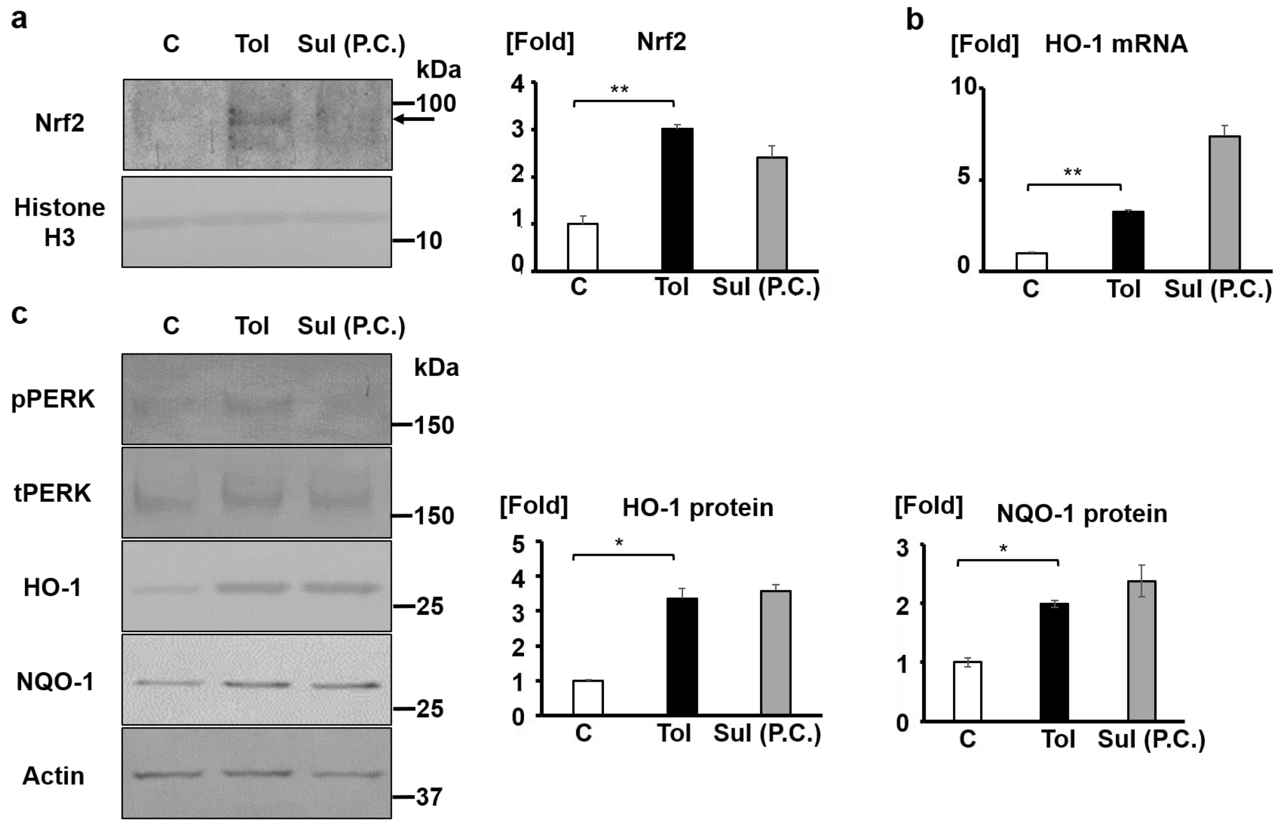

(a) Tolvaptan promotes Nrf2 nuclear translation in H9C2 cells. (Left panel) Western blotting of Nrf2 in nuclear extract. H9C2 cells were treated with 200 µM tolvaptan or 5 µM sulforaphane for 12h, following which nuclear fraction was separated using commercially available reagents for nuclear extraction. Arrow indicates the band of Nrf2. (Right panel) Densitometric analysis of Nrf2. Bar indicates average from three experiments. Tukey's test, ** $P < 0.01$. C: control (DMSO), Tol: 200 µM tolvaptan, Sul: 5 µM sulforaphane. P.C.: positive control.

(b) Tolvaptan increases HO-1 mRNA expression in H9C2 cells. H9C2 cells were treated with 200 µM tolvaptan for 4h, following which HO-1 mRNA expression was examined using qPCR. Bar indicates average from three experiments. Tukey's test, ** $P < 0.01$. C: control (DMSO), Tol: 200 µM tolvaptan, Sul: 5 µM sulforaphane. P.C.: positive control.

(c) Tolvaptan induces PERK phosphorylation and the protein expression of HO-1 and NQO-1 in H9C2 cells. (Left panel) H9C2 cells were treated with 200 µM tolvaptan or 5 µM sulforaphane for 12h. (Right panel) Densitometric analysis of HO-1 and NQO-1 are presented. Bar indicates average from three experiments. Tukey's test, * $P < 0.05$. C: control (DMSO), Tol: 200 µM tolvaptan, Sul: 5 µM sulforaphane. P.C.: positive control.

Supplementary Figure 2.

Tolvaptan does not activate the Nrf2/HO-1 antioxidant pathway in HK2 cells, which do not endogenously express V2R.

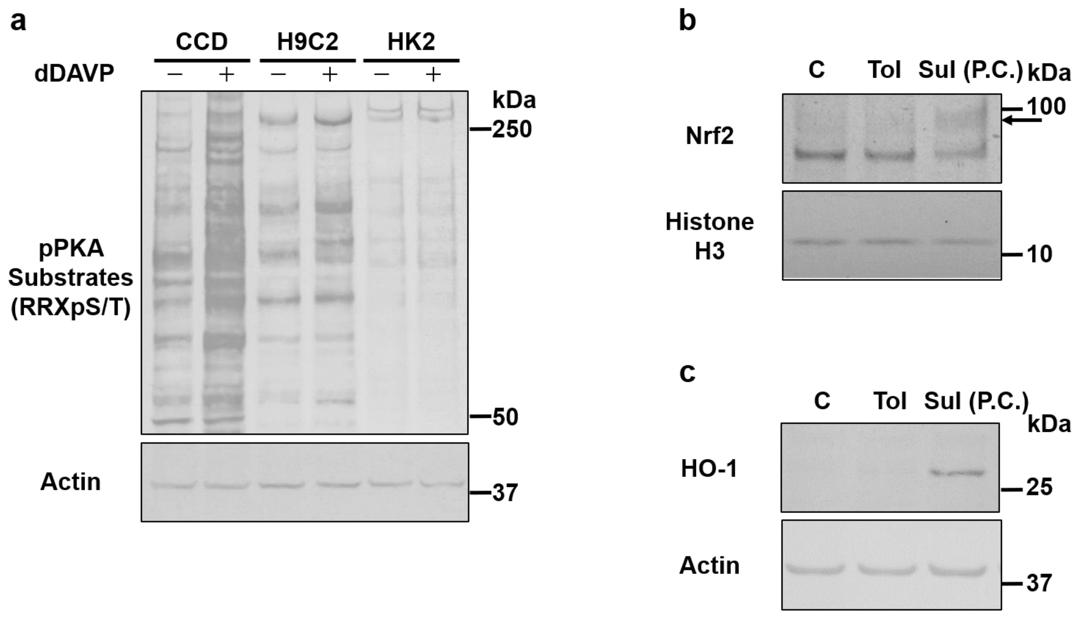

(a) A selective V2R agonist (dDAVP) does not activate cAMP/protein kinase A (PKA) signaling pathway in HK2 cells in contrast to V2R-expressing mpkCCD and H9C2 cells. Phospho-PKA substrate antibody was used to evaluate PKA activity. mpkCCD cells, H9C2 cells, and HK2 cells were treated with or without 1 nM dDAVP for 12h.

(b) Tolvaptan does not promote Nrf2 nuclear translation in HK2 cells. Western blotting of Nrf2 in HK2 nuclear extract. HK2 cells were treated with 200 μM tolvaptan or 5 μM sulforaphane for 12h, following which nuclear fraction was separated using commercially available reagents for nuclear extraction. Arrow indicates the band of Nrf2. C: control (DMSO), Tol: 200 μM tolvaptan, Sul: 5 μM sulforaphane. P.C.: positive control.

(c) Tolvaptan does not induce the protein expression of HO-1 in HK2 cells. HK2 cells were treated with 200 μM tolvaptan or 5 μM sulforaphane for 12h. C: control (DMSO), Tol: 200 μM tolvaptan, Sul: 5 μM sulforaphane. P.C.: positive control.

Supplementary Figure 3.

Uncropped gels of Figure 1.

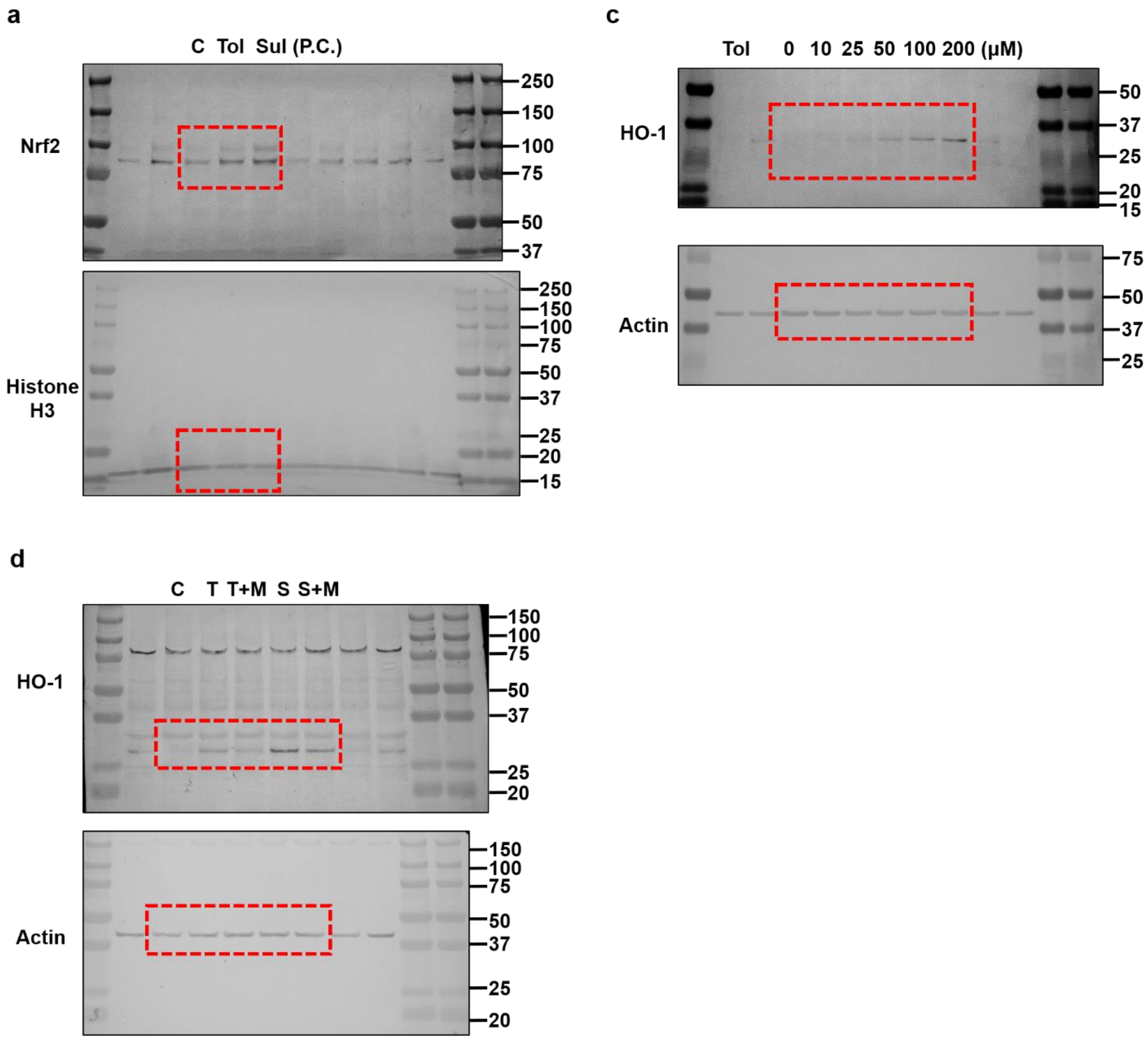

Uncropped gels of Figure 2.

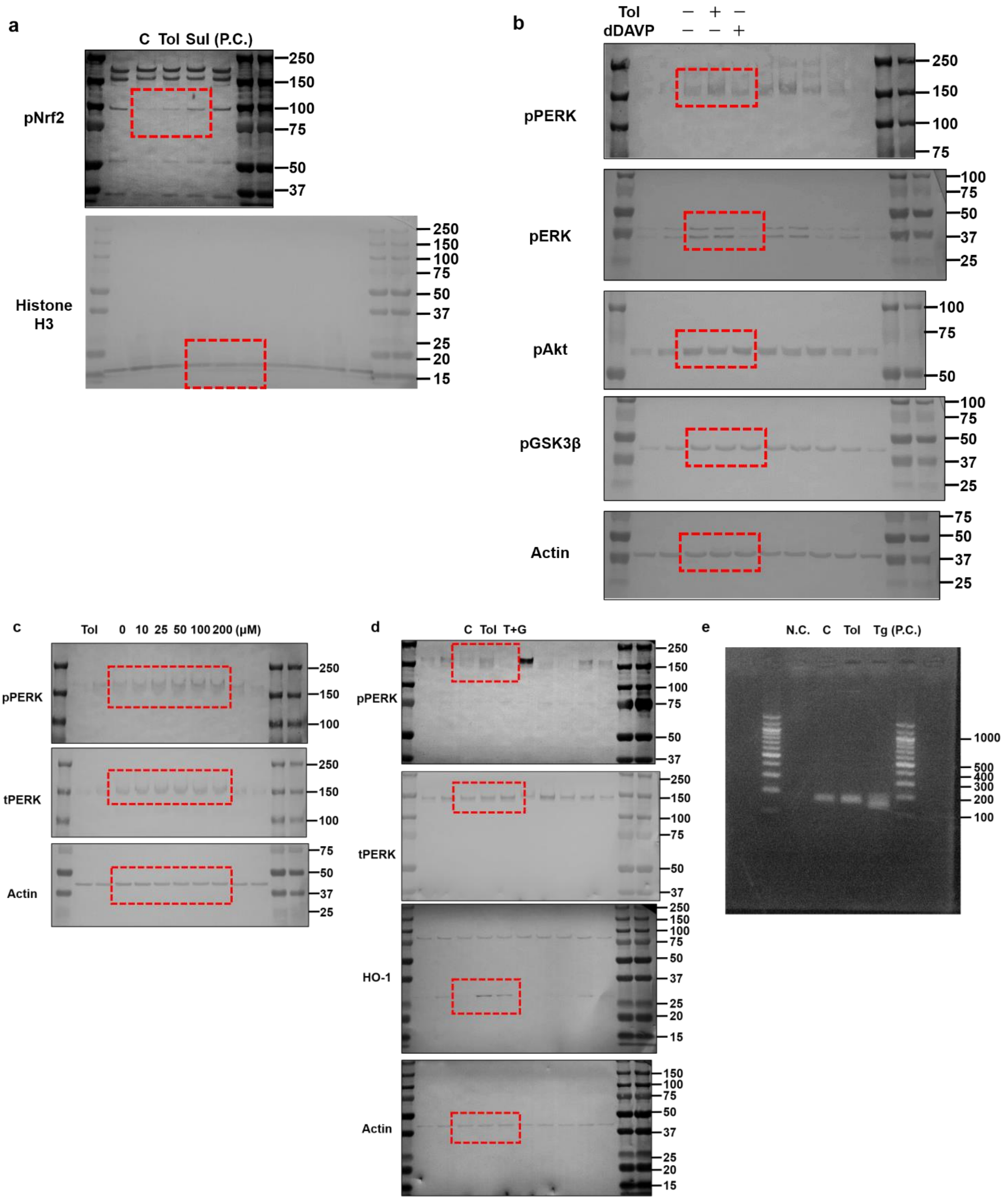

Uncropped gels of Figure 3.

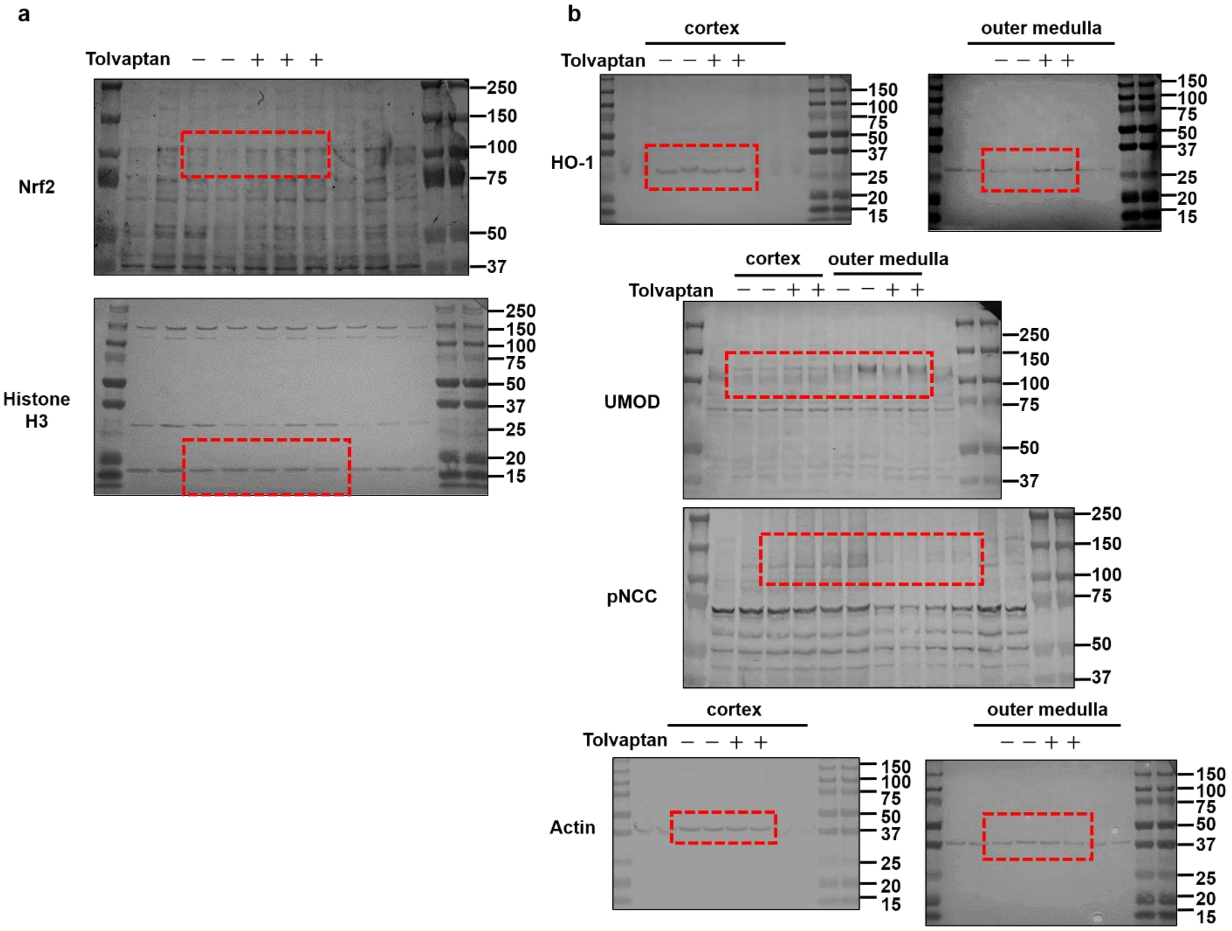

Uncropped gels of Figure 4.

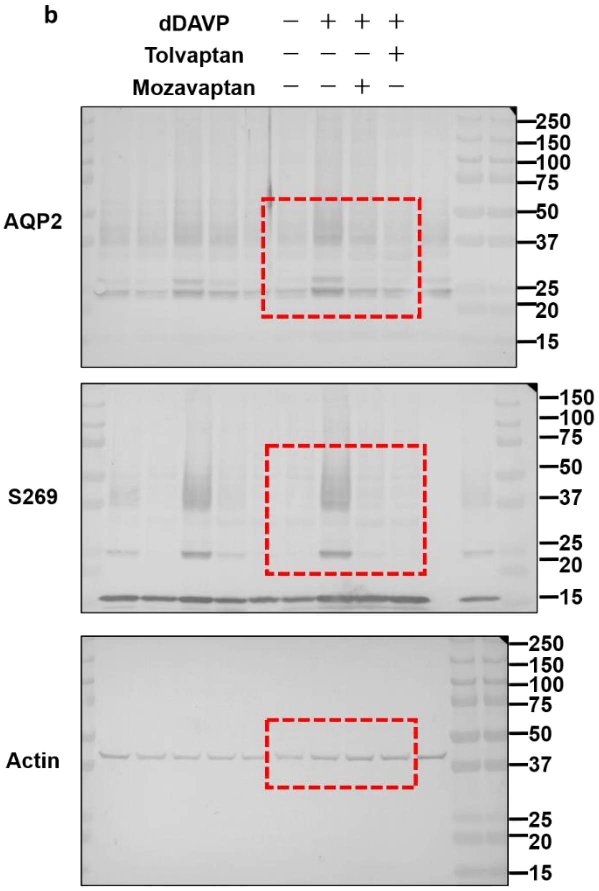

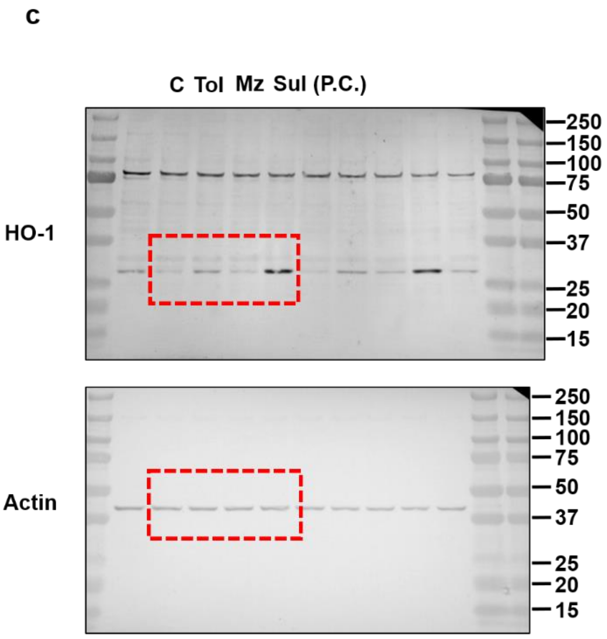

Uncropped gels of Figure 5.

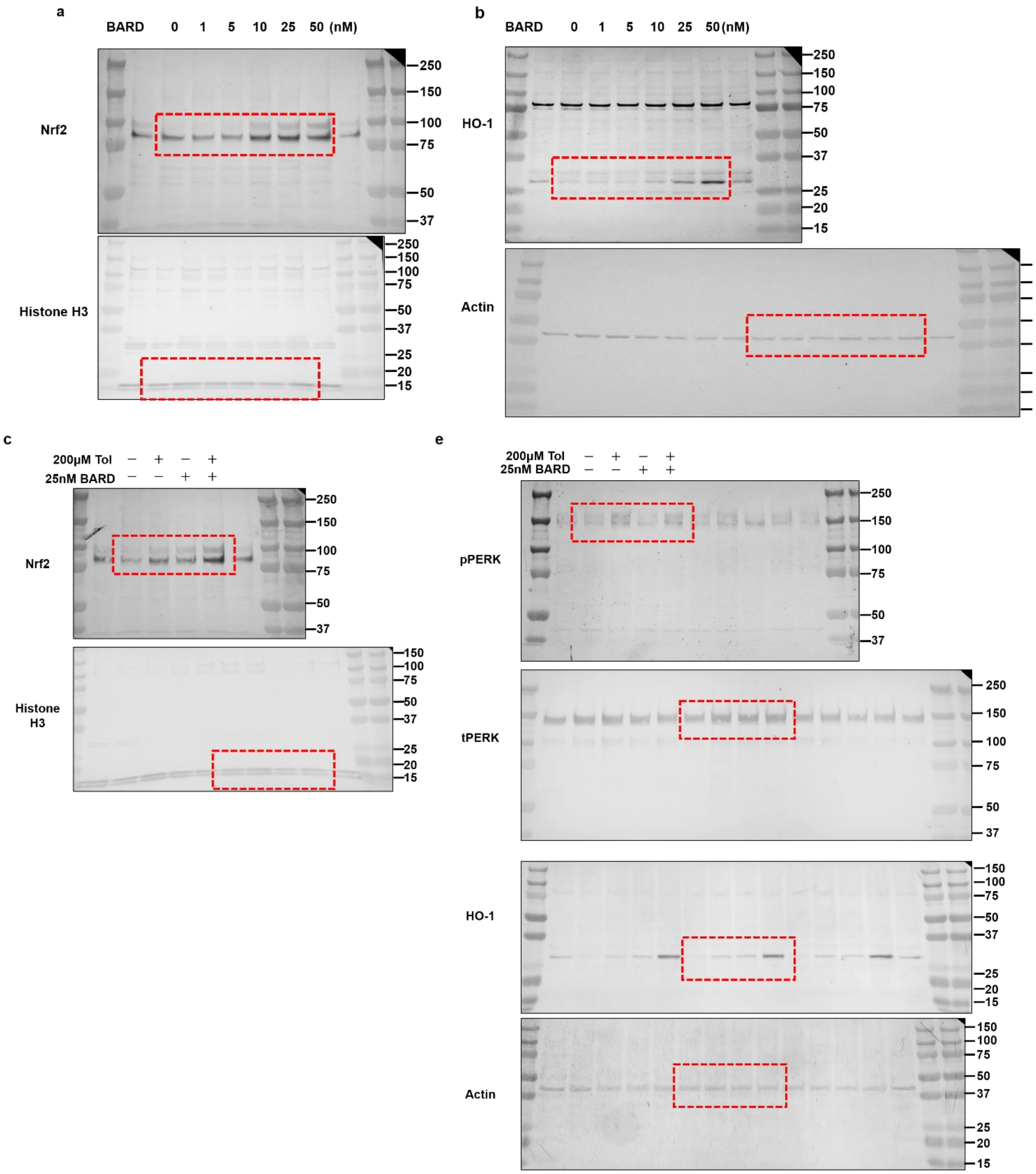

Uncropped gels of Supplementary Figure1.

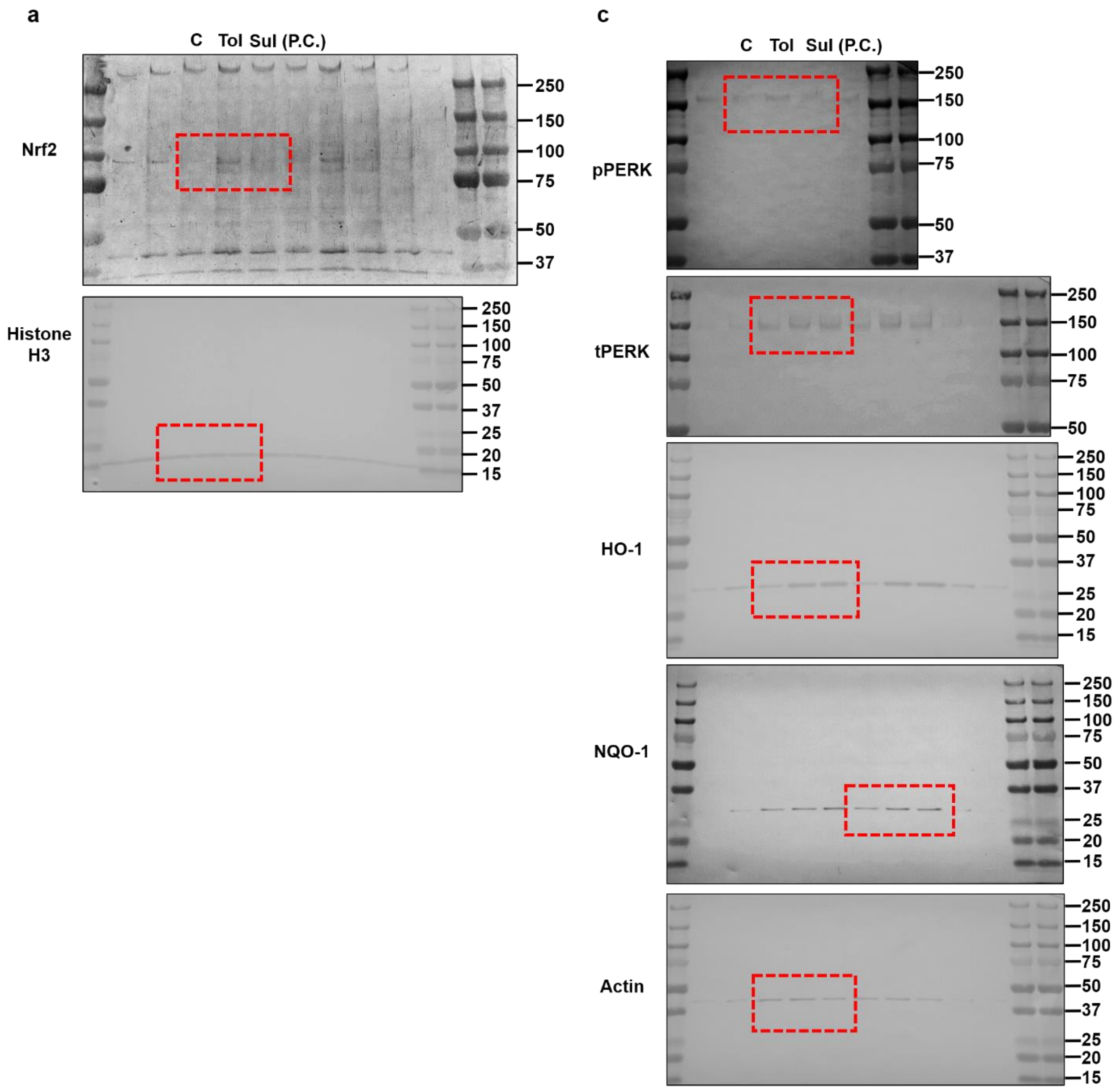

Uncropped gels of Supplementary Figure2.

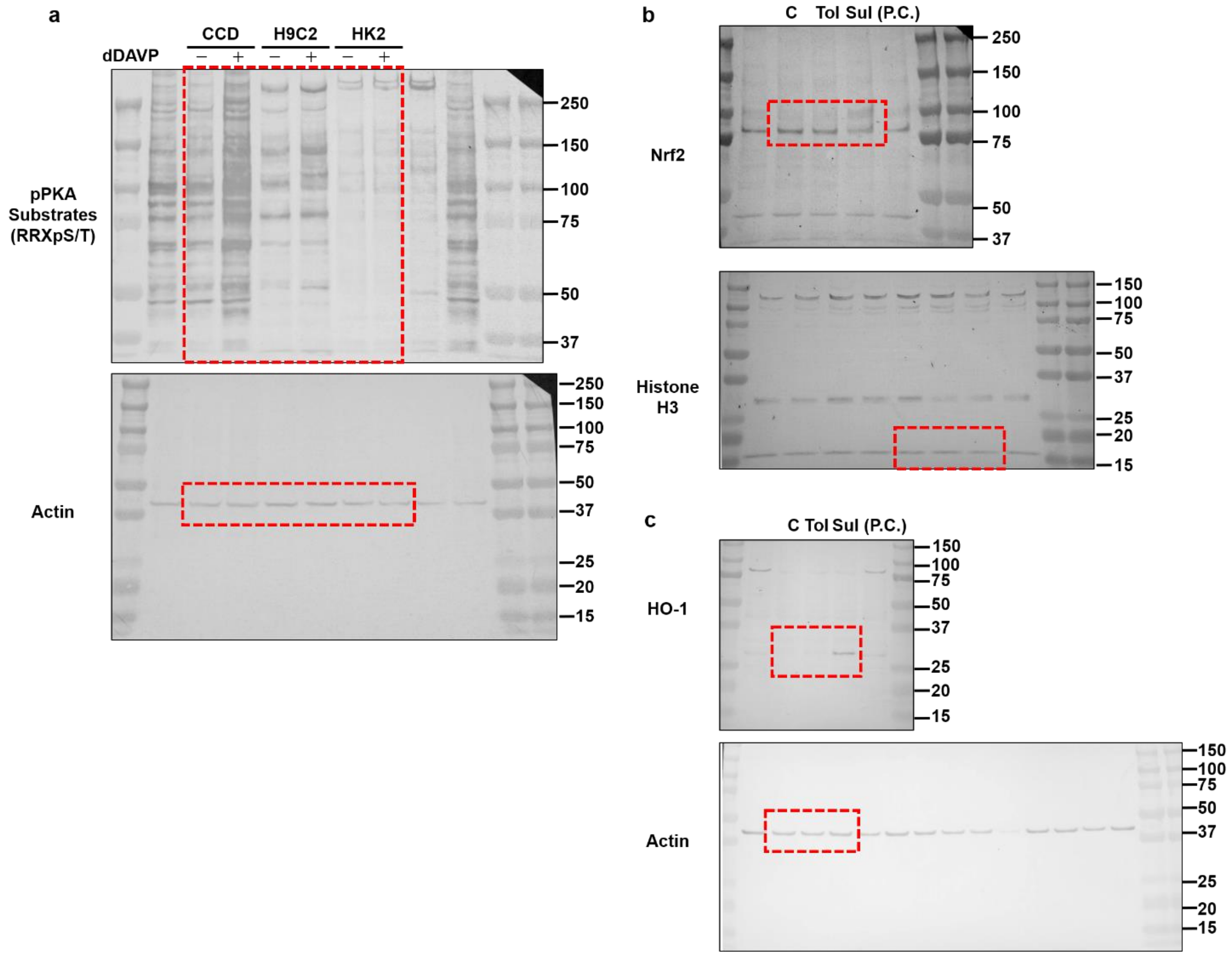

Supplement: Supplementary file 1 — Supplementary Figure [file 41598_2019_45539_MOESM1_ESM.pdf]
